# Optimization of the Mainzelliste software for fast privacy-preserving record linkage

**DOI:** 10.1186/s12967-020-02678-1

**Published:** 2021-01-15

**Authors:** Florens Rohde, Martin Franke, Ziad Sehili, Martin Lablans, Erhard Rahm

**Affiliations:** 1 Database Group, University of Leipzig, Leipzig, Germany; 2 Federated Information Systems, German Cancer Research Center, Heidelberg, Germany; 3 Complex Data Processing in Medical Informatics, University Medical Center Mannheim, Mannheim, Germany

**Keywords:** Mainzelliste, Privacy-preserving record linkage, Blocking, Locality-sensitive hashing

## Abstract

**Background:**

Data analysis for biomedical research often requires a record linkage step to identify records from multiple data sources referring to the same person. Due to the lack of unique personal identifiers across these sources, record linkage relies on the similarity of personal data such as first and last names or birth dates. However, the exchange of such identifying data with a third party, as is the case in record linkage, is generally subject to strict privacy requirements. This problem is addressed by privacy-preserving record linkage (PPRL) and pseudonymization services. Mainzelliste is an open-source record linkage and pseudonymization service used to carry out PPRL processes in real-world use cases.

**Methods:**

We evaluate the linkage quality and performance of the linkage process using several real and near-real datasets with different properties w.r.t. size and error-rate of matching records. We conduct a comparison between (plaintext) record linkage and PPRL based on encoded records (Bloom filters). Furthermore, since the Mainzelliste software offers no blocking mechanism, we extend it by phonetic blocking as well as novel blocking schemes based on locality-sensitive hashing (LSH) to improve runtime for both standard and privacy-preserving record linkage.

**Results:**

The Mainzelliste achieves high linkage quality for PPRL using field-level Bloom filters due to the use of an error-tolerant matching algorithm that can handle variances in names, in particular missing or transposed name compounds. However, due to the absence of blocking, the runtimes are unacceptable for real use cases with larger datasets. The newly implemented blocking approaches improve runtimes by orders of magnitude while retaining high linkage quality.

**Conclusion:**

We conduct the first comprehensive evaluation of the record linkage facilities of the Mainzelliste software and extend it with blocking methods to improve its runtime. We observed a very high linkage quality for both plaintext as well as encoded data even in the presence of errors. The provided blocking methods provide order of magnitude improvements regarding runtime performance thus facilitating the use in research projects with large datasets and many participants.

## Background

**Fig. 1:**
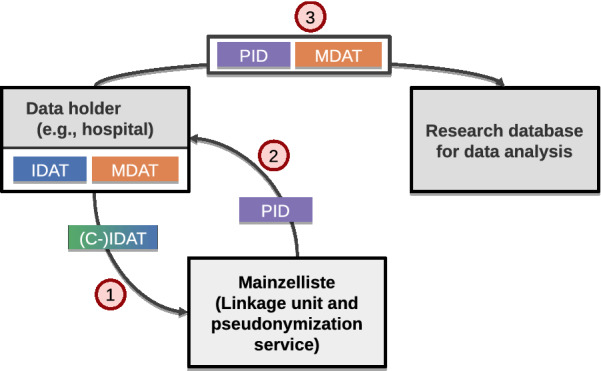
Linkage process with a centralized linkage unit (e.g., Mainzelliste)

Data analysis for biomedical research and clinical studies typically requires careful preparation and integration of relevant data from multiple data sources, in particular about patients who may have been treated in different hospitals and other institutions. Thus it is often required to identify records in different data sources referring to the same patients. This problem is known as record linkage and is necessary in most multi-site research efforts to handle since unique record identifiers are typically not available across different data sources. Record linkage relies on comparing personal identifying data, such as name and date of birth, of patients. Moreover, especially in the medical domain there are legal privacy requirements that generally do not allow to expose identifying data about patients to external parties thereby impeding the linkage of patient-related information. The latter challenge is addressed by privacy-preserving record linkage (PPRL) and pseudonymization techniques. PPRL has been an active area of research in the last decade and many protocols and methods have been proposed [1, 2]. The linkage of records is performed often by a trusted linkage unit that may also perform pseudonymization. For the sake of this article, we assume one unique pseudonym per patient. To fulfill the privacy requirements, each record is encoded or encrypted before linkage, in order to prevent an identification of individuals. Most recent PPRL strategies encode records by transforming identifying attributes into Bloom filters as proposed in [3]. Figure 1 illustrates the overall linkage process. At the data holders, we distinguish between two types of fields: identifying data (IDAT), needed for record linkage, such as name, date of birth and address, and medical data (MDAT), needed for data analysis, such as disease, blood pressure, medication etc. The linkage unit, e.g., Mainzelliste, only receives the IDAT values from the data holders but not the medical data to expose only minimal information for record linkage. The linkage unit determines whether new patient records match with previously provided records and returns the unique pseudonym (PID). Matching records, i.e., records referring to the same patient, will thus receive the same PID. After linkage, the data holders can associate the medical data (MDAT) with the respective PID and provide this information for data analysis. The PID values allow to combine medical information about the same patient from multiple sources, e.g., within a research database, without revealing sensitive IDAT information.

The sketched approach has to meet several requirements to be viable in practice. In particular, the approach should support multiple ($\ge 2$) data holders and provide high linkage quality so that all matching patients from different data holders are identified (high recall) and multiple records with the same PID indeed refer to the same person (high precision). Furthermore, the approach should be efficient and scalable, i.e., allow a fast matching and PID generation even for a very large number of records. Finally, a high degree of privacy should be maintained, in particular by supporting matching on encoded IDAT (C-IDAT) values. Thus, the linkage unit should never have access to unencoded sensitive information. Most proposed PPRL approaches only consider an one-time matching of two or more datasets (batch matching). However, they do not support the incremental matching of new records, which requires a suitable database to keep track of already matched records and their PIDs. Support for efficient incremental matching is often required in practice since previous linkage results can be accessed and updated.

### Mainzelliste

The Mainzelliste is a web-based open-source software for identity management [4]. Its core functionalities, pseudonymization and de-pseudonymization of patients, are accessible via a RESTful interface allowing self-explanatory usage via widely used web technologies. The pseudonymization process includes a configurable record linkage process, which by default uses an error-tolerant matching algorithm [5] to compute the similarity between pairs of records and find duplicates even in the presence of typos, interchanged fields, missing values etc.

Since its first release in 2013, Mainzelliste has been used by a constantly growing number of national medical research networks [6, 7], centralized biobanks [8], research platforms [9], commercial data capture and analysis suites [10, 11], registry software solutions [12, 13] and patient organizations and related disease registries [14, 15]. The software is under continuous development, incorporating community contributions from various research institutions [16].

Until now, however, there exists no detailed description of the Mainzelliste linkage process nor systematic evaluation of its match quality or runtime performance, leaving open its current potential and issues to be improved.

### Related work

The Mainzelliste can be used for conventional record linkage on original (plaintext) as well as for PPRL on encoded attribute values. A variety of other open-source record linkage tools exists [17], but most of them are limited to one-time batch matching. A comparison of the Mainzelliste with other tools for incremental matching on plaintext such as OpenEMPI was carried out in [4]. While PPRL has already been applied for several medical use cases in different organizations [18–20], to the best of our knowledge the Mainzelliste is the only publicly available PPRL tool with a RESTful web interface that has been used in a large number of real applications. In contrast to many other PPRL tools it is ready-to-use and easily deployable in medical applications rather than a prototype or library adding functionality to other programs. SOEMPI [21] builds on top of OpenEMPI and adds protocols for PPRL including encoding, matching and the exchange of the encoding secrets. The latter is necessary to ensure that all clients encode the IDAT in the same way. Such an exchange of parameters is not yet supported by the Mainzelliste which focuses on backend functionality. LSHDB [22] is a record similarity search system using parallel queries in distributed data stores for fast responses. However it does not assign matched records to a common PID and is designed to be used as a Java library instead of via a web interface. PRIMAT [23] is a toolbox providing many state-of-the-art encoding and matching techniques for PPRL including post-processing routines to achieve high linkage quality, but also lacks support for pseudonym management and web interfaces. All three tools provide blocking techniques to enhance the linkage performance, but focus on record-level Bloom filter in contrast to the field-level approach of the Mainzelliste (see below). Table 1 provides a comparison of the Mainzelliste and other open-source PPRL frameworks.

**Table 1:** Comparison of the Mainzelliste with other tools for Entity Resolution (ER) and PPRL

Product	Core Functionality	Incremental	Blocking	Usability
Mainzelliste	Identity Management with ER and PPRL	Yes	Soundex, LSH (Field-level Bloom filter) (our contribution)	RESTful web interface
Standard ER-Software	ER	Rarely	Yes	Library, Desktop Application
SOEMPI	PPRL	Yes	LSH (Record-level Bloom filter)	Web-Interface
LSHDB	PPRL	Yes	LSH (Record-level Bloom filter)	Library
PRIMAT	PPRL	Planned	LSH (Record-level Bloom filter)	Library, Desktop Application

## Objectives

We present the first detailed description of the Mainzelliste record linkage software, in particular the techniques and default settings used to match patient-related records. Moreover, we comprehensively evaluate the runtime and match quality of the Mainzelliste version 1.8. We comparatively evaluate record linkage based on original (plaintext) IDAT values against PPRL on encoded IDAT (C-IDAT) using field-level Bloom filters. We observed a poor runtime and scalability of the Mainzelliste since it misses support for blocking so that every new patient record has to be compared with every already known record. To improve runtimes, we extended the Mainzelliste to support phonetic blocking based on Soundex for the plain-text matching. For PPRL scenarios we also included blocking based on locality-sensitive hashing (LSH) that shows high efficiency and effectiveness in recent proposals [24]. However, LSH-based blocking has so far only been applied to record-level Bloom filter approaches, where all IDAT values are mapped into a single Bloom filter. Since the Mainzelliste utilizes field-level Bloom filter by default (see below), we have to adapt the standard LSH approach to work on multiple bit vectors. These optimizations were implemented within the Mainzelliste, but can be added to other PPRL tools as well. Finally, we evaluated our extensions, in particular the added blocking methods to identify suitable default parameter settings and to assess the improvements with respect to the previous implementation. Our key performance indicators were the execution time (runtime) for inserting a new patient to the Mainzelliste database as well as the standard linkage quality metrics recall, precision and F1-score.

## Methods

### Bloom filter encoding

The use of Bloom filters [25] for PPRL has been proposed by Schnell and colleagues [3] and has become the most popular encoding scheme for PPRL in research as well as in real applications [1, 2, 4, 18]. In general, identifying attributes are split into substrings of length *q* (*q*-grams) to build a set of record features $S = \{e_1, \dotsc , e_n \}$ that should be represented in a Bloom filter. The original strings can be surrounded by leading and trailing padding characters to ensure that all characters are included in the same number of *q*-grams. At first, a bit vector of size *m* is initialized with each bit set to zero. Moreover, *k* hash functions $h_1, \dotsc , h_k$ are defined and used to hash (map) the elements of *S* into the bit vector. Therefore *each* hash function is applied on *each* element of *S* and produces as output a position in the range $[0, m-1]$. Finally, the bits at the resulting positions are set to one. Setting a bit to one multiple times will have no effect. Given that identical q-grams are mapped to the same bit positions, a high overlap of q-grams leads to similar Bloom filters making them suitable for determining the record similarity e.g. using the Hamming Distance, the Jaccard index or the Dice coefficient (see Equation 4).

### Record linkage in the mainzelliste

**Fig. 2:**
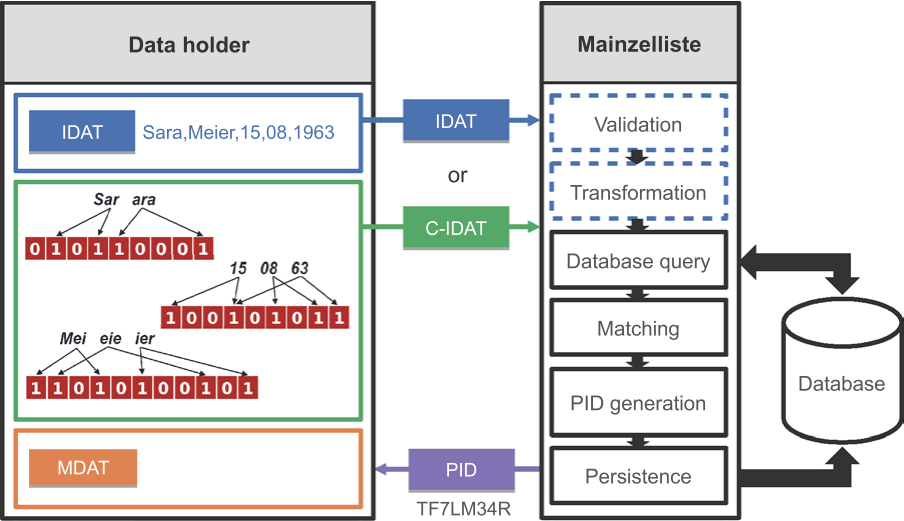
Workflow to process a new record in the Mainzelliste

In the following, we illustrate the process of adding a patient to the Mainzelliste as depicted in Figure 2. At first, the data holder sends the patient’s identifying data as HTTP request to the Mainzelliste server. The identifying data can be transmitted either as plaintext values, i.e., IDAT, or encoded as several field-level Bloom filters, i.e., C-IDAT.

Operating on IDAT, the Mainzelliste can execute a validation and transformation step before the actual linkage. Validity can be checked for attributes to identify errors, for instance invalid dates like 13-1990 (mm-yyyy). Furthermore, the data may be transformed into a standard form to facilitate the linkage process, e.g., remove diacritics and umlauts from names.

The actual record linkage process consists of several steps which are essentially the same for IDAT and C-IDAT. At this stage it is checked whether the record is already registered in the Mainzelliste. Therefore all previous records are retrieved from the database and matched with the query record *x* to find a possible duplicate. This matching is done by comparing the fields and computing an aggregated similarity score for each pair of records. In the next step (PID generation), a global identifier, a PID [26], is assigned to record *x*. If *x* is considered as a duplicate of a previously added record *y* then *y* is treated as representative for *x* and thus the PID of *y* is assigned to *x*. On the other hand, if *x* has no match, then a new PID is assigned to *x*. In both cases, the input request, i.e. record *x*, and the assigned PID are stored in the database (persistence).

Matching of two records *x* and *y* for both original and encoded data is based on their similarity *sim*(*x*, *y*) that has to exceed a certain threshold *t*. This similarity is determined as weighted sum of the similarities of all fields (attributes) $x_i$ and $y_i$ [5]:

1 $$\begin{aligned} sim(x,y)= \frac{\sum w_i \times sim(x_i, y_i)}{\sum w_i}, \end{aligned}$$

where the weight $w_{i}$ of the *i*th field is based on its average value frequency $f_{i}$ and error-rate $e_i$:

2 $$\begin{aligned} w_i= \log _2 \frac{(1-e_i)}{f_i} \end{aligned}$$

Table 2 shows the default weights of the Mainzelliste for German person data that originate from the evaluation of a German cancer registry and will also be used in our evaluation. The values reflect the discriminatory power of the different fields for matching.

Depending on the data type of the fields several similarity functions can be used for comparison. For string fields the Mainzelliste applies the Dice similarity based on the amount of overlapping q-grams, i.e., substrings of length q, where $q = 2$ is set by default (bi-grams). The Dice similarity can be calculated as

3 $$\begin{aligned} sim(x_i, y_i)= \frac{2 \times |q(x_i) \cap q(y_i)|}{|q(x_i)| + |q(y_i)|} \end{aligned}$$

where *q*(*s*) is the q-gram set of a string value *s*.

Numerical fields, e.g., day, month or year of birth, are compared by value equality. Hence, the similarity value is either 0 (unequal) or 1 (equal).

For comparing encoded fields (field-level Bloom filters) the Dice similarity is also used:

4 $$\begin{aligned} sim(x_i,y_i)= \frac{2 \times {{\,\mathrm{card}\,}}(x_i \wedge y_i)}{{{\,\mathrm{card}\,}}(x_i) + {{\,\mathrm{card}\,}}(y_i)} \end{aligned}$$

where ${{\,\mathrm{card}\,}}(b)$ is the number of bits set to 1 in a Bloom filter *b* and $\wedge$ denotes the bitwise AND operation.

**Table 2:** Default field weights of the Mainzelliste

Attribute	*f*	*e*	*w*
First name	0.000235	0.01	12.04
Last name	0.0000271	0.008	15.15
Day of birth	0.0333	0.005	4.9
Month of birth	0.0833	0.002	3.58
Year of birth	0.0286	0.004	5.12
Date of birth	0.00007	0.005	13.8

The match classification of compared pairs uses two thresholds $t_1, t_2$, with $t_1 > t_2$. A pair of records *x* and *y* is considered as:

- Match $$\Leftrightarrow sim(x,y)\ge t_1$$
- Possible Match $$\Leftrightarrow t_2 \le sim(x,y) < t_1$$
- Non Match $$\Leftrightarrow sim(x,y) < t_2$$

In principal, one record *x* can match to more than one other record. For example, assuming $t_1 = 0.8$, *x* can match to $y_1$ with a similarity score of 0.9 and to $y_2$ with a similarity score of 0.95. The Mainzelliste therefore adopts a *best-match* selection strategy, i.e., only the record with the highest similarity score is considered as match.

The class of possible matches is used for records where a definite match decision is not possible. In practice, possible matches could be manually verified by a domain expert. In the rest of this paper, we set $t_1 = t_2$ and thus consider only definite matches.

### Standard blocking

A potential performance problem of record linkage with Mainzelliste is that comparing a record with all records in the database leads to poor scalability since the number of comparisons increases with more data. Blocking is a common technique to reduce the number of match comparisons [27]. The standard blocking approach partitions the records according to a function on the values of selected fields, returning blocking keys. The similarity computation for matching is then restricted to pairs of records from the same partition, i.e., records sharing the same blocking key.

### Phonetic blocking

A frequently used blocking approach for matching of unencoded data is phonetic blocking, e.g., based on the Soundex function [28]. Phonetic encoding functions, like Soundex, are typically applied on name attributes and aim to produce the same output for input values with a similar pronunciation (even with typographical variations or errors). For instance, the Soundex value for both names ’Sara’ and ’Sara**h**’ is S600. However, since the first letter of the attribute value is preserved in the Soundex code, typographical variations at the beginning of a name, e.g., ’Zarah’ (Z600) vs. ’Sarah’ (S600), can not be compensated. Such problems can be reduced by choosing several blocking functions, e.g., Soundex for both first name and last name.

### LSH-based blocking

Locality-sensitive hashing (LSH) was proposed for solving the nearest neighborhood problem in high-dimensional data spaces [29]. The basic idea of LSH is to apply a set of hash functions on the objects of interests, e.g., bit vectors. These hash functions are sensitive to a certain distance measure *d*, e.g., Hamming or Jaccard distance. Each hash function has the property that the probability of a collision, i.e., same output value for two different input value is much higher for objects with a small distance (high similarity) than for objects with greater distance (low similarity). Please note that the hash functions used for LSH are completely different from those used to construct Bloom filters.

LSH can be utilized as blocking approach for PPRL using bit vectors (Bloom filters) [30]. For this purpose hash functions that are sensitive to the Hamming distance can be used (HLSH). These functions $f_i$ return the bit value at position *i* in the bit vector [30]. For instance, applying the function $f_7$ on the bit vector $\mathtt {11011001}$ would return the bit value on position 7 and therefore 1. In order to group similar records, a blocking key is constructed by using $\Psi$ such hash functions which are selected randomly. Then, the output values of these $\Psi$ hash functions are concatenated to obtain the blocking key.

As a consequence, the parameter $\Psi$ represents the length of the blocking key, i.e., number of selected bits. Due to the probabilistic nature of LSH, it is possible that two bit vectors with a small distance (high similarity) may produce different blocking keys, namely if the bit value(s) at one or several of the $\Psi$ positions are different. To improve the error tolerance, $\Lambda$ blocking keys are therefore generated to increase the probability that two similar but different bit vectors agree in at least one blocking key so that the encoded records are compared with each other to decide about whether they match.

**Fig. 3:**
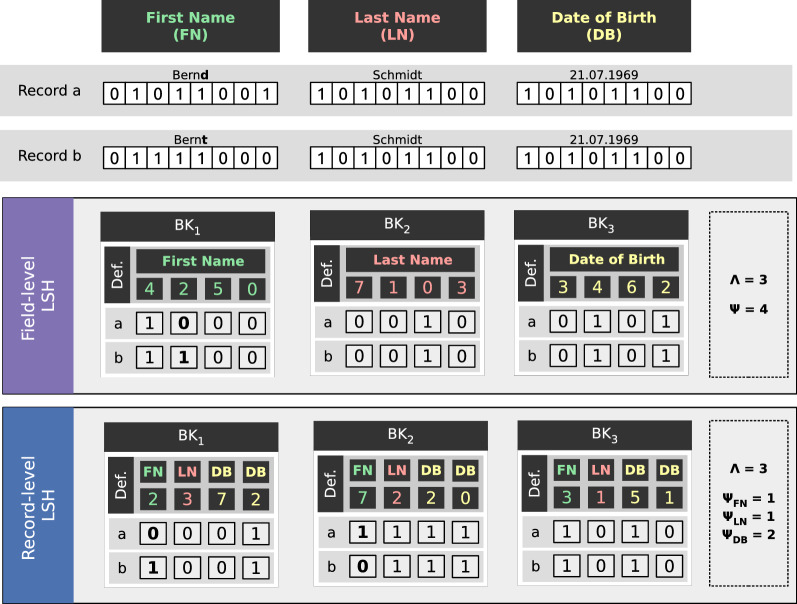
LSH-blocking variants on field-level Bloom filter

The two LSH parameters $\Psi$ and $\Lambda$ need to be carefully selected. A higher value for $\Psi$ increases the probability that only bit vectors with a high similarity are assigned to the same block. Hence, a higher $\Psi$ will lead to smaller blocks and thus fewer intra-block comparisons. On the other hand a lower $\Psi$ will instead produce larger blocks but also decreases the probability that two similar bit vectors are missed due to erroneous data. On the other hand, the higher $\Lambda$, the higher is the probability that two similar bit vectors share one blocking key. However, at the same time, the number of blocks and thus the number of candidates that need to be processed increases leading to increased execution time.

### LSH-based blocking on FBFs

LSH has been used as a blocking method for PPRL in several approaches [24, 31]. However, LSH-based blocking has so far only been applied to record-level Bloom filters where a single bit vector represents all identifying data of a person. In contrast, the Mainzelliste has focused on field-level Bloom filters (FBF) as they promise higher linkage quality which is strictly required in most medical contexts. The LSH-based blocking method thus needs to be modified as it has to operate on multiple input Bloom filters instead of only a single one. In the following, we propose two methods to apply LSH on a set of field-level Bloom filters $\{ {bv}_1, \dotsc , {bv}_p \}$ where *p* denotes the number of Bloom filters (fields) used for blocking. Figure 3 shows an example of these methods.

#### Field-level LSH

As a first approach, we consider a field-dependent selection strategy, where a certain number $\Lambda _i$, $i \in \{1, \dotsc , p\}$, of LSH blocking keys is constructed for each field separately. All bits of a single LSH key are drawn from the same FBF and hence each key is affected by exactly one field. For the example of Figure 3, we have chosen a single key of length 4 for each of the three considered fields. The two sample records have the same blocking key for two of the three keys.

The main benefit of this approach is that it is error-tolerant even if several field values are different or missing. At least one matching field is sufficient to assign two records into the same block. On the other hand, as each blocking key solely depends on a single FBF, the resulting blocks can become large when there are only few different field values or frequent field values, e.g., popular last or first names.

#### Record-level LSH

We also consider a field-independent selection strategy. For each LSH blocking key ${bk}_i$ with $i \in \{1, \dotsc , \Lambda \}$ we select a certain number $\{\Psi _1, \dotsc , \Psi _p \}$ of positions from each FBF. As a consequence, the $\Psi = \sum _{i=1}^p \Psi _i$ bits of each LSH key will be drawn from different FBFs. For the example of Figure 3, we have again $\Lambda = 3$ blocking keys of length $\Psi =4$ but the bits are selected from all three fields (1 bit each from the first two fields and 2 bits from the third field). Only the third key has the same value for the two considered records.

In contrast to the field-level LSH approach, the record-level strategy can lead to smaller blocks as each LSH blocking key depends on several FBFs and thus fields. Therefore the record-level LSH strategy is assumed to produce less candidates and consequently less record pair comparisons. However, the record-level strategy may also be less error-tolerant than the field-level strategy. In particular, if attributes are erroneous or contain missing values, then the probability that these attributes will affect several or even all LSH blocking keys increases. As a consequence, such cases can lead to missing matches (false-negatives). Therefore, more LSH keys may be needed to avoid or limit this problem.

#### Treatment of compound fields

Duplicate patient records differing in small details, e.g. typos, can be matched by error-tolerant algorithms. However, real-world records of the same patient can also differ significantly, e.g. if one has only simple first or last names while the other contains several first names (or one first and a middle name) or double last names, e.g. due to marriage. For plaintext data, the record linkage algorithm of the Mainzelliste can be configured to split such compound names on hyphens and whitespace. The calculation of the overall similarity of two compound fields can then be determined per component. For example, compound-sensitive matching would yield a similarity value of 1 for the comparison of last name *“Pinkett Smith”* with *“Pinkett”* (instead of 0.5).

We implemented a similar approach for encoded matching using field-level Bloom filters. This is achieved by an additional preprocessing step to create multiple Bloom filters for compound field values. Matching and blocking is then performed for each of the component Bloom filters.

#### Implementation as database-side blocking

**Fig. 4:**
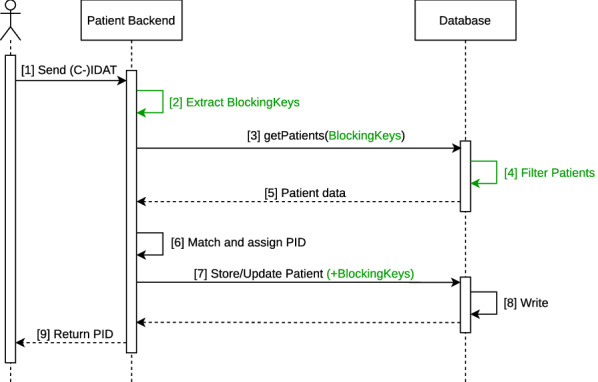
Communication within the Mainzelliste between the backend logic and the database before (black) and after our contributions (green)

The Mainzelliste uses a database to store the patient identifiers. The duration of adding a new patient mainly depends on the database query for candidates and the subsequent matching. In the original implementation without blocking *all* patients are retrieved from the database. A subsequent blocking would significantly reduce the number of comparisons and thereby the matching time. However the unnecessary query of most patient records should also be avoided. Therefore we implement a database-side blocking to improve the runtime of both subprocesses. Fig. 4 illustrates how our contributions are integrated into the interactions within the Mainzelliste backend, specifically for the communication between the patient processing logic and the database. After receiving a new request the Mainzelliste determines blocking keys for this record according to the configured blocking method (e.g., Soundex for plaintext or a LSH method for Bloom filter) (step 2). These blocking keys are passed to the database when retrieving the matching candidates (3). The database uses these keys to select and return only those stored patients that share at least one key (4). The matching step (6) is not altered as the filtering of the patients is already conducted within the database. For new or updated patients the backend submits the blocking keys to the database along the patient data (7) to allow inclusion of the patient in future queries.

### Evaluation

The goal of the evaluation is to comparatively analyze match quality and runtime performance for both plaintext and encoded field values for both the original Mainzelliste and the changed version. Furthermore, we want to analyze the impact of the proposed blocking strategies.

#### Datasets

For the evaluation we use one real world and four synthetically generated, near-real datasets each with the fields first name, last name and date of birth. Table 3 shows main features of the five datasets, in particular their sizes and error rates.

**Table 3:**
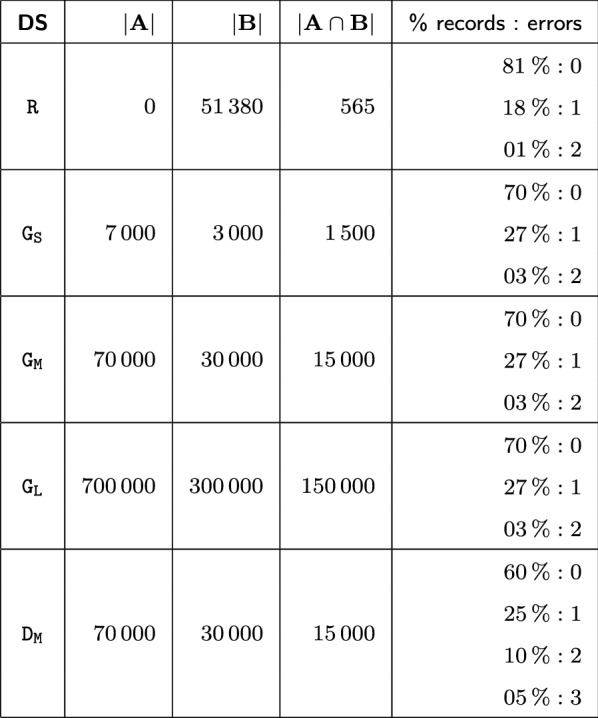
Description of the datasets, each with the size of the initial patient list |*A*|, the number of inserted patients |*B*|, the number of duplicate records $|A \cap B|$ and the proportion of records with a certain amount of erroneous fields

Dataset $\mathtt {R}$ is based on a real-world dataset with approximately $50\,000$ person records that were drawn from the civil register of a German city. This dataset is of high quality and contains only 565 duplicate records. An analysis of the duplicates shows that approximately $80\,\%$ are equal in all of their fields, but the remaining duplicates contain missing values, diacritics and multiple names in first and last name fields. All records of dataset $\mathtt {R}$ are sequentially inserted so that each additional record is matched against the records already stored in the Mainzelliste database.

To systematically evaluate the impact of the dataset size and data quality, we synthetically generated four additional datasets with near-real person names derived from look-up files and frequency distributions from German census data. For this purpose, we employ a customized version of the GeCo data generation and corruption tool used in previous research on record linkage [32]. The $\mathtt {G}$ datasets are generated in three sizes to evaluate the scalability of the linkage: small, medium, and large with $10\,000$, $100\,000$ and $1\,000\,000$ records in total. For these datasets we assume that a subset A of $70\%$ of the records are already inserted in the Mainzelliste database and that the records of the remaining subset B are added (matched and inserted) one by one. For the large dataset $\mathtt {G_L}$ the runtimes without blocking were already too high so that we only evaluate it for a randomly selected subset of B encompassing 10% of its records. The quality of the $\mathtt {G_L}$ datasets is lower than for the real dataset $\mathtt{R}$ since we assume a relatively high share of duplicate records (50% of the records in subset B). Furthermore, 30% of the duplicates are assumed to contain one or two erroneous field values as indicated in the last column of Table 3.

For quality evaluation, we additionally consider the “dirty” dataset $\mathtt {D_M}$. Dataset $\mathtt {D_M}$ has the same size than $\mathtt {G_M}$ but more errors, e.g., phonetic variation, OCR errors and typos, that are introduced by GeCo’s corruption component. In $\mathtt {D_M}$ $40\,\%$ of the duplicate records are erroneous including $5\,\%$ with errors in all three fields to provide a pessimistic scenario for achieving high match quality.

#### Bloom filter encoding

Bloom-filter-based record linkage requires the preprocessing steps to be done before the actual encoding and therefore by the data holder. Table 4 shows the data cleaning methods used for each field. For dataset $\mathtt {R}$ an additional step was performed to split compound fields as described above. After preprocessing, all fields are split into bigrams that are mapped into the Bloom filters. The three components of the birthday have been encoded in a joint Bloom filter. An essential parameter for encoding is the ratio of the number of hash functions to the length of the Bloom filter. The larger the ratio, the more bits are set on average in the bit vector. The applied encoding parameters shown in Table 4 result in an average share of approximately 25% 1-bits.

**Table 4:**
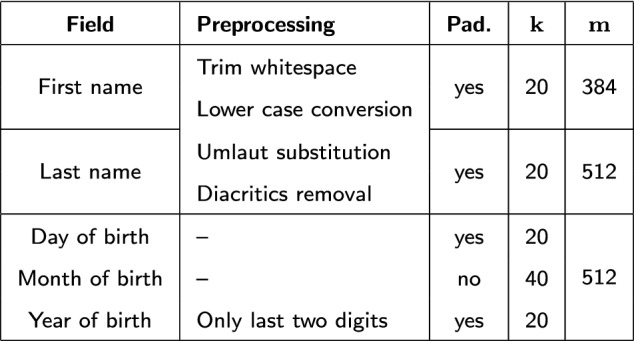
Bloom filter encoding used for the evaluation with *k* as the number of hash functions and *m* as the length of the Bloom filter

#### Evaluation metrics

We use the standard metrics recall, precision and F1-score to evaluate linkage quality. Recall measures the proportion of found true matches from all true matches. Precision measures the proportion of true matches from all found matches. The F1-score is the harmonic mean of these two metrics.

$$\begin{aligned} \text {Recall}&= \frac{\#\text {TruePositives}}{\#\text {TruePositives} + \#\text {FalseNegatives}}\\ \text {Precision}&= \frac{\#\text {TruePositives}}{\#\text {TruePositives} + \#\text {FalsePositives}}\\ \text {F1-score}&= \frac{2 \cdot \text {Recall} \cdot \text {Precision}}{\text {Recall} + \text {Precision}} \end{aligned}$$

Runtime for inserting patients is measured within the Mainzelliste and therefore it does not include the network latency (delay) of the HTTP requests. Please note that the time for inserting a patient includes the retrieval of records from the database, the actual matching as well as the time needed for persistence.

Furthermore, we determine the average number of candidates for each record and calculate the reduction ratio (RR) which is defined as the proportion of comparisons that is evaded by the use of blocking:

5 $$\begin{aligned} \text {RR} = 1 - \frac{\#\text {candidates with blocking}}{\#\text {records in database}} \end{aligned}$$

For example, a value RR=0.999 (99.9%) refers to a reduction of the number of comparisons by a factor of 1,000.

#### Blocking parameters

**Fig. 5:**
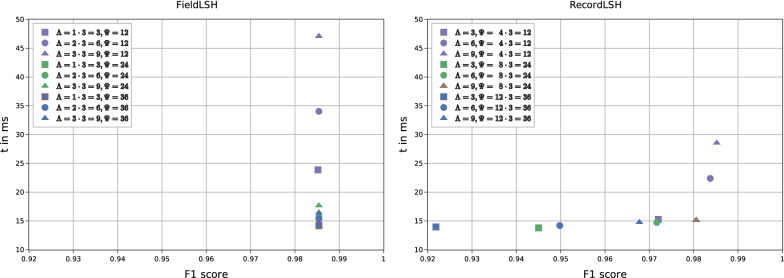
F1 score against runtime for different numbers of LSH keys ($\Lambda$) and LSH key lengths ($\Psi$) determined for dataset $\mathtt {G_M}$

For blocking on plaintext fields we use two Soundex codes on first and last name. As a result two records are compared if they share the same Soundex value for either the first or the last name. LSH blocking requires the configuration of the two parameters $\Lambda$ and $\Psi$ (number and length of blocking keys). We therefore evaluated different settings on dataset $\mathtt {G_M}$ to determine suitable default parameters for each LSH method. Fig. 5 shows the obtained F1-score and runtime results for different values for $\Lambda$ and $\Psi$. For FieldLSH (left part of Fig. 5) the F1-scores are very stable as at least one of the three fields per record is error-free for $\mathtt {G_M}$. We therefore chose $\Lambda = 3$, corresponding to one key per field and $\Psi = 36$ as it results in short runtimes. However for RecordLSH (right part of Fig. 5) a higher number of blocking keys $\Lambda = 9$ and shorter keys with $\Psi = 24$, i.e., 8 hashes for each field ($8 \cdot 3 = 24$), yield a good compromise between linkage quality and runtime.

Additionally, we apply the key restriction approach proposed in [24] to exclude bit positions that are frequently set to 0 or 1 and they can cause larger block sizes. The bit frequencies are determined at runtime based on the first 1000 inserted records and a prune ratio of 0.5 is applied.

#### Matching parameters

To determine a suitable threshold to maximize the F1-score of the linkage result without blocking, we systematically evaluated different threshold settings $t = \{ 0.8, 0.85, 0.9, 0.95 \}$. For dataset $\mathtt {R}$, we apply the threshold $t = 0.9$ for plaintext matching and $t_{BF} = 0.95$ for PPRL with Bloom filters. For the more erroneous datasets $\mathtt {G_*}$ and $\mathtt {D_M}$ we set $t = 0.8$ and $t_{BF} = 0.85$.

#### Benchmark setup

All experiments are conducted on a desktop computer equipped with an Intel i7-6700, 32 GB main memory and a SSD running Ubuntu 18.04, MySQL 5.7 and Tomcat 8.5.

## Results and discussion

Table 5 shows the results of all evaluations for the five datasets and without and with Bloom Filters (BF), without and with (Soundex or LSH) blocking. Rows without blocking correspond to the original implementation of the Mainzelliste whereas rows with blocking represent the respective results with our improvements. For each of the five configurations per dataset, the table shows the linkage quality results (recall, precision, F1 score) as well as the average (insert) runtime per record, the number of blocks, the number of match candidates and the achieved reduction ratios.

**Table 5:**
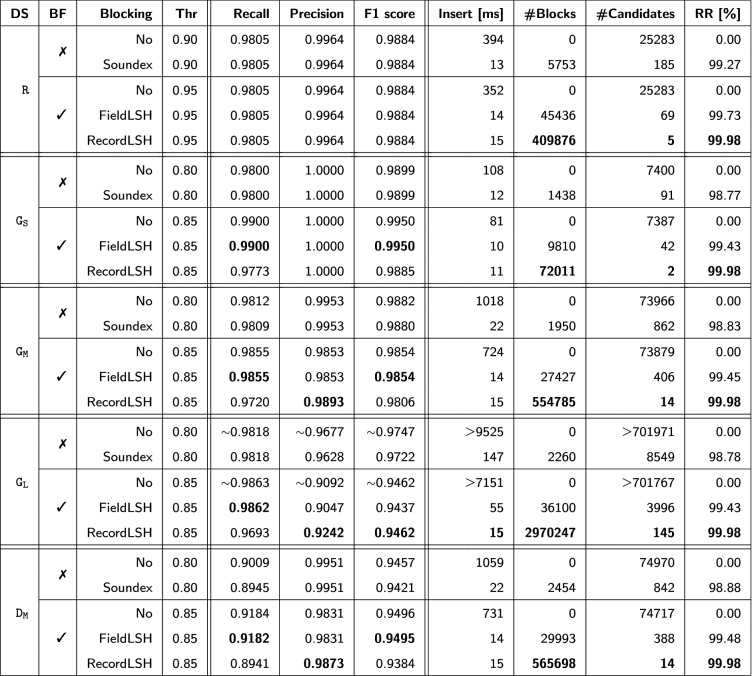
Evaluation results

### Comparison of plaintext and encoded matching without blocking

The evaluation results of the original Mainzelliste (rows in Table 5 without blocking) show excellent linkage quality for both plaintext matching and PPRL using Bloom filter for the real dataset R and the small and medium sized datasets $\mathtt {G_S}$ and $\mathtt {G_M}$. For these datasets precision values of almost 100% and F1-scores of about 99% are achieved. This has been made possible by the error-tolerant matching approaches. For the real dataset $\mathtt {R}$ the match support for compound names also proved essential. The results in Table 6 show that the special treatment for compound names improves recall from 89% for plaintext matching and 92% for Bloom filter matching to 98% and a corresponding improvement of the F1-score to almost 99%. The execution time is generally faster for Bloom Filter matching than using plaintext data, e.g., for dataset $\mathtt {G_M}$ by almost 30%, since the similarity computation for bit vectors is faster than for string values.

**Table 6:** Comparison of quality metrics for dataset $\mathtt {R}$ with and without the use of compound fields (CF) and Bloom filters (BF)

**BF**	**CF**	**Threshold**	**Recall**	**Precision**	**F1 score**
✗	✗	0.90	0.8920	0.9980	0.9420
✗	✓	0.90	0.9805	0.9964	0.9884
✓	✗	0.90	0.9204	0.9980	0.9515
✓	✓	0.95	0.9805	0.9964	0.9884

Linkage quality is somewhat reduced for the dirtier dataset $\mathtt {D_M}$ (to about 95% F1 score) and the large dataset $\mathtt {G_L}$ (to 94.6-97.5% F1 score) for both plaintext and Bloom filter matching. For $\mathtt {D_M}$, the high precision is retained but recall is decreased since the increased error rates lead to lower similarity for duplicate records that are partially missed for the default thresholds. A reduced threshold would improve recall at the expense of a lower precision which is considered more harmful since it could lead to consider different persons as matches. For the large dataset $\mathtt {G_L}$, however, we observe a decrease in precision for the default threshold values since there are many more match candidates than for $\mathtt {G_M}$ leading to more wrong match decisions. In this respect, Bloom filter matching achieves a lower precision (about 0.91) compared to plaintext matching (0.97). We believe that the problem can be reduced by an optimized configuration, e.g. using additional fields for matching and longer bit vectors, but a more detailed analysis is beyond of this paper.

### Impact of the proposed blocking methods

**Fig. 6:**
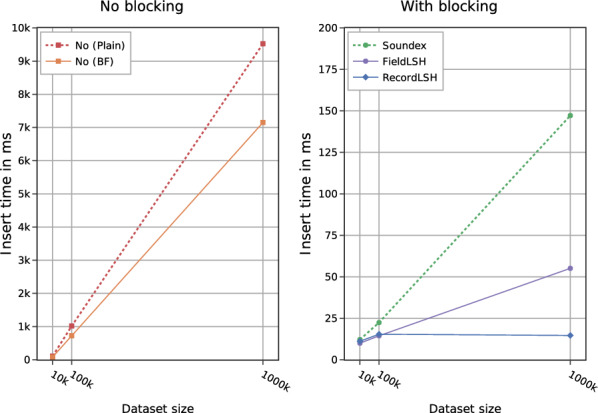
Comparison of average insertion times per patient on datasets $\mathtt {G_S}$, $\mathtt {G_M}$ and $\mathtt {G_L}$ without (left) and with (right) blocking

The newly introduced blocking methods lead to dramatic improvements in the runtime of the Mainzelliste software by several orders of magnitude. Figure 6 illustrates the average insert time per record vs. the dataset size. In the original implementation without blocking (left part of Fig. 6) these excution times rise linearly with the number of records. This leads to an unacceptably long runtime per record for dataset $\mathtt {G_L}$ of up to 9.5 (7) seconds for plaintext (Bloom filter) matching and thus to execution times of more than one month for 300.000 records. Applying blocking (right part of Fig. 6 with different scaling of the y axis) leads to drastically improved execution times, e.g. by a factor of almost 500 using RecordLSH on dataset $\mathtt {G_L}$. Moreover, runtimes are stable for RecordLSH on datasets of different size. FieldLSH and especially Soundex are more dependent on the data volume and experience an increase in runtimes with more records. This is because their number of blocks increases only modestly with more data so that the average size of blocks and thus the number of comparisons per record increase with larger data volumes. Still for dataset $\mathtt {G_L}$ the execution time for blocking with FieldLSH (Soundex) is a factor of 130 (65) faster than without blocking. The reduction ratios achieve even better values of up to 99.98%, i.e. a factor 5000 in the number of comparisons.

These high runtime improvements are achieved without reduction in linkage quality as can be seen from the F1 score values in Table 5. There are some relatively small differences between the two LSH variants. FieldLSH leads to larger blocks than RecordLSH thereby enabling a slightly better recall. On the other hand, the smaller blocks of RecordLSH favor a better precision, especially for the large dataset $\mathtt {G_L}$. RecordLSH is much faster than FieldLSH for the large dataset $\mathtt {G_L}$, but the runtimes are almost the same (actually slightly worse) for the smaller datasets. This is because the reported insert time are only partially determined by the match time but also include the time to store new records and their blocking keys into the database. The latter persistence step needs slightly more time for RecordLSH than for FieldLSH because of the higher number of LSH blocking keys (9 vs. 3).

Given the comparable linkage quality and runtimes for both FieldLSH and RecordLSH in most cases, we recommend FieldLSH as the default blocking strategy for the Mainzelliste except for very large datasets. This is because it is much easier to configure than RecordLSH and a simple approach with a single blocking key per field proved to perform very well.

## Conclusions

We presented an evaluation of the Mainzelliste software for privacy-preserving record linkage with regard to its linkage quality and runtime performance. We also developed and analyzed an optimized version of the software for fast execution times. Our results using real-world and near-real datasets showed mostly excellent linkage quality for both standard (plaintext) and privacy-preserving matching using field-level Bloom filters. However the previous implementation showed poor runtime performance and limited scalability as new records have to be compared with all previously known records. The new version of the software includes Soundex blocking for plaintext matching and two new variants of LSH blocking at the field level. These methods drastically improve the runtime without reducing linkage quality and can also be used by other PPRL tools as they are not specific to the Mainzelliste. Our improvements have been integrated into the official source code repository of the Mainzelliste and will be made available with the upcoming release of version 1.9.

## Data Availability

The datasets used during the evaluation are available in the Zenodo repository, https://zenodo.org/record/3695363.
